# Stress induced cardiomyopathy presenting as acute coronary syndrome: Tako-Tsubo in Mercogliano, Southern Italy

**DOI:** 10.1186/1476-7120-5-36

**Published:** 2007-10-16

**Authors:** F Cangella, A Medolla, G De Fazio, C Iuliano, N Curcio, L Salemme, G Mottola, Marco Agrusta

**Affiliations:** 1 Cardiology Department, 'Montevergine' Cardilogy Clinic, Mercogliano, Italy; 2 Casa di Cura Montevergine, Via Mario Malzoni, 1, 83013 Mercogliano (Av.), Italy

## Abstract

**Background:**

Tako-tsubo syndrome (TTS) in its typical (apical) and atypical (non-apical) forms is being increasingly recognized in the West owing to early systematic coronary angiography in acute coronary syndromes (ACS).

**Aim of the study:**

To assess the incidence, the clinical characteristics and the outcome of TTS in a single high volume cath lab in Southern Italy over the last 6 years.

**Methods:**

Among 1674 consecutive patients (pts) referred to our coronary care units in the last 6 years (2001–2006) for ACS we selected 6 (0.5%) pts (6 women; age 57 ± 6 years) who fulfilled the following 4 criteria: 1) transient left ventricular wall motion abnormalities resulting in ballooning at contrast ventricolographic or echocardiographic evaluation; 2) normal coronary artery on coronary angiography performed 5 ± 9 hours from hospitalization; 3) new electrocardiographic ischemic-like abnormalities (either ST-segment elevation or T-wave inversion) and 4) emotional or physical trigger event.

**Results:**

At admission all pts had presumptive diagnosis of ACS and ECG revealed ST elevation in 3 (50%) and T wave inversion with QT elongation in 3 (50%). In the acute phase cardiogenic shock occurred in 2 (33%) and heart failure in 1(16%). Presenting symptoms were chest pain in 6 (100%), dyspnoea in 2 (33%) and lipotimia in 1 (16%). At echocardiographic-ventricolographic assessment, the mechanical dysfunction (ballooning) was apical in all 6 pts ("classic" TTS). In all patients wall motion abnormalities completely reversed within 4.5 ± 1.5 days. The region of initial recovery was the anterior and lateral wall in 4 cases and the lateral wall in 2 cases. Ejection fraction was 35 ± 8% in the acute phase and increased progressively at discharge (55 ± 6%) and at 41 ± 20 months follow-up (60 ± 4%, p < 0.001 vs. baseline). All patients remained asymptomatic with minimal (aspirin, beta blockers, antihypertensive and antidislipidemic therapy) treatment.

**Conclusion:**

Classic TTS is a frequent serendipitous diagnosis after coronary angiography showed "surprisingly" normal findings in a clinical setting mimicking an ACS. Despite its long-term good prognosis life threatening complications in the acute phase can occur.

## Introduction

Stress induced cardiomyopathy is described as an acute cardiomyopathy characterized by acute, but rapidly reversible, left ventricle systolic dysfunction in the absence of atherosclerotic coronary artery disease which appears to be triggered by intense psychological and physical stress [1]. This syndrome has been firstly described in Japan in 1990 by Sato et al, who proposed the term 'Tako-Tsubo', the name used by Japanese fishermen for the fishing pot with a narrow neck and a wide base that is used to trap octopus. In fact, the left ventricle with Tako-tsubo cardiomyopathy shows the peculiar appearance of a rounded bottom an a narrow neck on the end-systolic left ventriculogram [2]. Various definitions have been proposed in order to describe this syndrome such as apical ballooning syndrome (ABS), broken heart syndrome and stress or ampulla cardiomyopathy [2]. The exact incidence of ABS appears to be unclear, it ranges form 1 to 2% of patients who present, at admission, a clinical scenario which often mimics acute myocardial infarction. The occurrence of ABS syndrome seems to be significantly recurrent in postmenopausal women although it has been described also in younger women and males. The most frequent finding on the admission ECG is a mild ST-segment elevation which appears to occur in approximately 50–60% of the patients [2]. The 12-lead ECG alone in not helpful in differentiating ABS from ST-elevation myocardial infarction.

Characteristic evolutionary changes that occur over 2 to 3 days include resolution of the ST-segment elevation and subsequent development of diffuse and often deep T-wave inversion that involves most leads. New pathological Q waves may occasionally be observed and, furthermore, it has been possible to observe a prolongation of the corrected QT interval [2]. Although there are rare exceptions, the proposed Echocardiographic Mayo Clinic Criteria for the clinical diagnosis of ABS underline a transient apical regional wall motion abnormalities, usually extending beyond a single epicardial vascular distribution. Most, if not all of the patients, appear to have a mild increase of cardiac biomarkers. Typically, no obstructive coronary artery disease has been found. Although the pathogenesis still remains unclear, a common element appears to be an exaggerated sympathetic activation. The mechanism underlying the association between sympathetic stimulation and myocardial stunning in unknown. One possibility is ischemia resulting from epicardial coronary arterial spasm. An alternative mechanism is microvascular spasm. A third possible mechanism is direct myocyte injury [3]. Activation of cardiac adrenoreceptors is experimentally proven to induce left ventricular (apical) dysfunction. In fact, there is evidence that apical myocardium has enhanced responsiveness to symphatetic stimulation [4], potentially making the apex more vulnerable to sudden surges in circulating catecholamine levels. Aim of the present study was to describe a case series of 6 ABS observed in the last 6 years in a tertiary care referral center with high volume cardiac cath lab in Mercogliano, Southern Italy.

## Methods

During the last 6 years (2001–2006), among 1674 consecutive patients (pts) referred to our coronary care units for ACS, 6 patients (age 57 ± 6 years; all women) presenting at admission with acute onset of cardiovascular event have been retrospectively selected. All fulfilledthe following 4 predetermined criteria to define Tako-Tsubo cardiomyopathy [2]: 1) transientleft ventricularwall motion abnormalities resulting in ballooning at contrast ventricolographic or echocardiographic evaluation; 2) normal coronary artery on coronary angiography (performed 5 ± 9 hours from the hospitalization); 3) new electrocardiographic ischemic-like abnormalities (either ST-segment elevation or T-wave inversion) and 4) emotional or physical trigger event.

All patients underwent, at admission, two-dimensional transthoracic echocardiography, 12 lead electrocardiogram, serial measurements of cardiac isoenzymes including creatine kinase, creatine kinase MB fraction, and troponin. Transthoracic echocardiography has again been repeated at discharge. All patients have been followed-up for a median period of 40 months.

### Echocardiographic data

All patients underwent comprehensive transthoracic echocardiography examination at rest, upon discharge and at a median period of 40 months follow-up. End-systolic, end-diastolic volumes, ejection fraction and wall motion score index (each segment scored from 1 = normal/hyperkinetic, to 4 = dyskinetic, in a 16 segment model of the left ventricle) were calculated following the recommendations of the American Society of Echocardiography [5]. The left ventricular volumes and ejection fraction were measured by modified biplane Simpson's method and adjusted for body surface area. The systolic pulmonary artery pressure has been derived from maximal velocity of tricuspid Doppler tracing adding the value of the right atrial pressure. The right atrial pressure was estimated on the basis of inspiratory collapse index of the inferior vena cava. Diastolic function was determined from the pattern of mitral and pulmonary venous flow velocity by pulsed Doppler echocardiography, complemented by mitral annular velocity by tissue Doppler imaging, when needed [5]. Diastolic dysfunction was staged as being "absent" (grade 0), "mild" (grade 1, impaired relaxation), "moderate" (grade 2, pseudo-normalized filling pattern), and "severe" (grade 3, restrictive filling pattern). All echocardiographic examinations were performed using commercially available instruments with a cardiac probe (2.5–3.5 MHz): Acuson Sequoia (Mountain View, Ca), Esaote Mylab (Genoa, Italy).

### Statistical analysis

Values are expressed as mean ± standard deviation (SD) unless indicated otherwise. Groups were compared by parametric or non-parametric tests (t-tests and Wilcoxon-Mann-Whitney tests, respectively). More than 2 groups were compared using the analysis of variance. Post-hoc tests were performed (if significant differences were proved globally) with the help of Newman-Keuls test. A p < 0.05 was considered as statistically significant.

## Results

**Figure 1:**
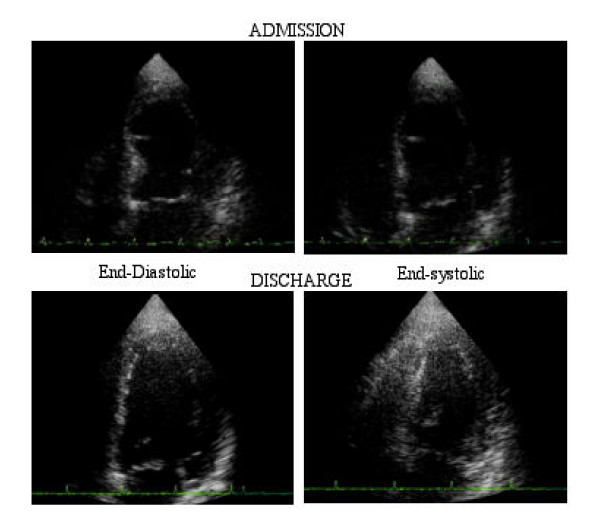
A typical echocardiographic appearance of apical ballooning syndrome. Left up: end-diastolic image on admission and left down end-diastolic image at discharge; right up: end-systolic image on admission and right down: end-systolic image at discharge.

Demographic and clinical features of the study patients are reported in Table 1. All patients reported a physical and/or an emotional distress in the hours immediately before admission consisting of death of a relative in 4, strong financial loss in 1 and heated argument with daughter in 1. The presenting symptoms were chest pain in 6, dyspnea in 2, lipotimia in 1 and severe dyspnea with acute heart failure in 1 patient. The initial electrocardiogram showed sinus rhythm in all patients with ABS, with a median heart rate of 105 beats per minute; 3 patients had ST-segment elevation of at least 1 mm, and 3 patients had diffuse T-wave inversion; 3 patients had a prolonged QT interval corrected for heart rate (QTc). Q waves did not appear in any of the observed patients. On admission, echocardiogram showed an apical dysfunction in all of the patients. Over time, there was a normalization of regional wall motion (Figure 1), a reduction of left ventricular end-systolic volume and improvement of diastolic function (Table 2).

**Table 1:** Clinical and Electrocardiographic features

Age (years)	57 ± 6
Sex (Male/Female)	0/6
Smoking habit, n (%)	0
Hypercholesterolemia, n (%)	2 (33.3%)
Hypertension, n (%)	3 (50%)
Diabetes mellitus, n (%)	1 (16.6%)
Chest pain/Dyspnea	6/2
Menopause, n (%)	5 (83.3%)
CK (U/l)	196.2 ± 91
CK-MB (U/L)	19.5 ± 5.6
CK normalization (days)	2.6 ± 1.2
Troponin (ng/ml)	14.5 ± 1.2
Mioglobin (ng/ml)	78.6 ± 36.2
White blood cells/μl	11866 ± 4500
ESR	26.8 ± 16
C-reactive protein	20.3 ± 13.4
Heart Rate (beats/min)	110 ± 13
ST – elevation	3 (50%)
T-wave inversion	3 (50%)
ST/T modification Peripheral leads	3 (50%)
ST/T modification Precordial leads	6 (100%)
Long QT	3 (50%)
Q wave	0

**Table 2:** Echocardiographic findings

	**On admission**	**Pre-discharge**	**Follow-up**
EDV mL	144.1 ± 46.6	116.6 ± 45.8*	96.8 ± 36.9*
ESV mL	92.8 ± 36.3	52.7 ± 21.1*	36.6 ± 14.1*
EF(%)	35 ± 8	55 ± 6*	60 ± 4*
WMSI	1.7 ± 0.1	1 ± 0**	1 ± 0**
Diastolic dysfunction ≥ moderate n (%)	3 (50%)	0 **	0*
PAPS (mmHg)	30.6 ± 3.8	28.7 ± 2	27.3 ± 2.2

EDV = end-diastolic volume; ESV = end-systolic volume; PAPS = Pulmonary artery arterial pressure; WMSI = Wall Motion Score Index

* p < .05 vs. baseline; ** p < 0.01 vs. baseline

**Figure 2:**
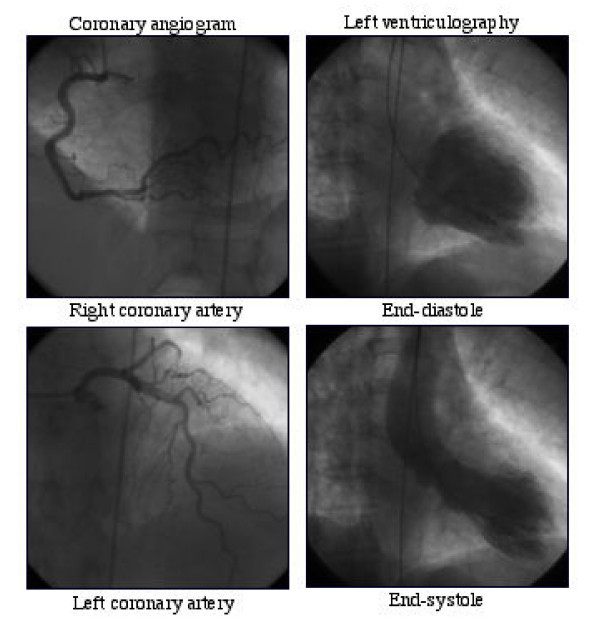
A typical angiographic appearance of apical ballooning syndrome with normal coronary arteries.

Emergency coronary angiography showed angiographically normal coronary arteries in all with ventriculographic confirmation of apical ballooning observed by echocardiogram (Figure 2). Due to acute refractory heart failure, intraortic balloon pump was used in 2 patients. At ventriculography all of the patients showed apical akinesis and normokinesis of middle and basal segments. All patients were uneventfully discharged and remained asymptomatic at follow – up (median 44, range 12–68 months). At follow-up echocardiogram, normal regional and global left ventricular systolic and diastolic function were restored (Table 2).

## Discussion

**Figure 3:**
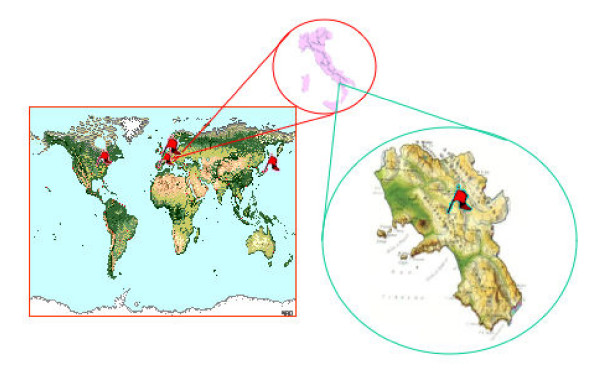
Geographic origin of published reports and spotlight on Tako-Tsubo in Mercogliano, Southern Italy.

We described 6 cases of tako-tsubo cardiomyopathy in a region of Southern Italy close to Naples. In the contemporary definition and classification of the Cardiomyopathies proposed in 2006 by the American Heart Association, stress ("Tako-Tsubo") cardiomyopathy is classified among acquired forms of cardiomyopathy [6]. A recent systematic review on apical ballooning syndrome identified 14 case series published on international peer-reviewed literature, totalling about 200 patients [7]. Our data are broadly consistent with these previous reports, regarding epidemiological profile, clinical presentation and long-term follow-up. ABS accounts 2% of acute coronary syndromes, with most cases occurring in post-menopausal women. Ischemic-like ST-T changes most frequently occur with mild elevation of cardiac biomarkers. The acute left ventricular dysfunction typically involves the apex, although recently some apex-sparing variants of the syndrome have been described [8]. The acute left ventricular dysfunction usually recovers in a few days or weeks, and long-term follow-up is usually uneventful. Of note, the geographic origin of published reports originates from Japan (9 studies), USA (3 studies), Belgium and Spain (1 study each) [9]. In the present case series, we describe 6 cases all originating from Irpinia, a region of Southern Italy, near Naples but with different ethnic origin and lifestyles (Figure 3). This report again emphasizes that ABS can be much more diffuse than previously thought, and it can be easily recognized, if one thinks of it and applies clearly pre-defined criteria.

Clinicians should consider this syndrome in the differential diagnosis of acute coronary syndromes, especially in post-menopausal women with a recent history of acute emotional or physical stress. The non-invasive suspicion rests on the typical echocardiographic appearance apical ballooning – usually reversible within a few days or weeks. The angiographic documentation of normal coronary arteries is necessary to substantiate the diagnosis.

## List of Abbreviations

ABS: Apical ballooning syndrome

ACS: Acute coronary syndrome

Pt: patient

TTS: Tako-tsubo syndrome
